# Association of MTHFR C677T polymorphism with severity and localization of chronic atrophic gastritis patients without *Helicobacter pylori* infection: a case control study

**DOI:** 10.1186/s12885-020-07208-2

**Published:** 2020-08-05

**Authors:** Siya Kong, Feng Ye, Yini Dang, Yifei Hua, Guoxin Zhang

**Affiliations:** 1 Department of Gastroenterology, First Affiliated Hospital of Nanjing Medical University, No. 300 Guangzhou Road, Nanjing, 210029 China; 2 First Clinical Medical College of Nanjing Medical University, Nanjing, China

**Keywords:** MTHFR C677T, Polymorphism, Atrophic gastritis, Homocysteine, Incisura

## Abstract

**Background:**

Previous reports indicate that the methylenetetrahydrofolate reductase (MTHFR) 677C > T polymorphism plays a role in gastric cancer. However, whether it influences the development and progression of atrophic gastritis remains ambiguous. We aimed to determine the possible relationship between MTHFR C677T polymorphism and the severity of atrophic gastritis.

**Methods:**

A total of 128 patients without *Helicobacter pylori* infection were included in the study. The severity of gastric atrophy was assessed by pathological diagnosis using OLGA and OLGIM Gastritis Staging System. MTHFR 677C > T genotyping was performed by digital fluorescence molecular hybridization. Categorical variables were analyzed by percentages using the χ^2^ test.

**Results:**

In this study, the TT genotype was significantly more frequent among *Helicobacter pylori*-negative patients aged ≤44 years (age ≤ 44 years vs. > 44 years, *P* = 0.039). Patients with TT genotype showed a higher ratio of incisura with atrophy or intestinal metaplasia (TT vs. CC + CT, *P* = 0.02). Furthermore, TT genotype was associated with more severe lesions compared with the CC + CT genotypes (TT vs. CC + CT for atrophy: *P* = 0.07; for intestinal metaplasia: *P* = 0.01; for moderate-to-severe lesions: *P* = 0.01). OLGA and OLGIM stages III-IV were observed more frequently in patients with TT genotype compared with CC + CT genotypes (for OLGA: *P* = 0.003; for OLGIM: *P* = 0.036).

**Conclusions:**

The MTHFR 677C > T TT genotype showed an increased risk of moderate-to-severe lesions by OLGA and OLGIM stages, and these results indicate that MTHFR C677T polymorphism may act as a predictive marker for precancerous gastric lesions, especially in *Helicobacter pylori*-negative patients aged ≤44 years.

## Background

Gastric cancer is the third primary cause of cancer-related death in the world [1]. According to the Correa’s theory, a series of precancerous lesions (atrophic gastritis [AG], intestinal metaplasia [IM], and dysplasia) is caused by chronic inflammation of the gastric mucosa, which leads to the evolution of stomach cancer [2]. AG is a chronic disorder losing the oxyntic glands, which is characterized by lamina propria fibrosis or their replacement with pseudo-pyloric or IM [3]. Therefore, early supervision of AG could reduce the incidence of gastric cancer [4].

Cancer development is a result of intricate interactions between genetic and environmental factors. Epigenetic changes like DNA methylation play an important part in cancer development [5]. 5,10-Methylenetetrahydrofolate reductase (MTHFR) has a role in folate metabolism and is associated with DNA, RNA, and protein methylation [6]. MTHFR C677T polymorphism is associated with various tumors, as this change in sequence reduces the activity of this enzyme [6, 7]. Indeed, individuals with the TT and CT genotypes have mildly higher homocysteine levels than CC homozygotes [8]. In addition, hyperhomocysteinemia is a growing risk for different precancerous lesions according to its observed effects on morbidity and mortality among patients [9, 10]. Previous studies have shown that gastric diseases might cause hyperhomocysteinemia through nutrient malabsorption [11]. As a result, AG patients with TT genotype have a much higher risk of gastric cancer. Therefore, MTHFR C677T polymorphism might be useful in predicting the development and severity of gastrointestinal cancer, especially in Asian populations [12–14]. However, the function of MTHFR C677T polymorphism in stomach precancerous lesions is still unclear. Therefore, based on the risk levels (0-IV) ranked by the Operative Link on Gastritis Assessment (OLGA) and Operative Link on Gastric Intestinal Metaplasia Assessment (OLGIM) grading systems [15], we investigated the relationship between MTHFR C677T polymorphism and AG in *Helicobacter pylori*-negative patients in this study.

## Methods

### Trial design and subjects

This study was designed as a single-center, cross sectional observational trial. Consecutive patients who underwent endoscopy were recruited in the First Affiliated Hospital of Nanjing Medical University from November 2018 to December 2019. A total of 128 patients suffering from AG were diagnosed for the first time and had not received any previous treatments, and each diagnosis was confirmed after endoscopy by pathological examination. The exclusion criteria were: (1) *Helicobacter pylori* (*H. pylori*) positivity (an independent contributing factor to the development of AG); (2) previous eradication treatment of *H. pylori*; (3) use of proton pump inhibitors, antibiotics, or H_2_-receptor blockers in the previous 6 months; and (4) intake of drugs influencing the level of folic acid. We assessed *H. pylori* infection by the ^13^C-urea breath test (UBT). The study protocol was reviewed and approved by the ethics committee of the First Affiliated Hospital of Nanjing Medical University. We obtained written informed consent from every participant. This trial was completed and registered with ClinicalTrials.gov (ChiCTR1900020815,Chinese Clinical Trial Registry).

### Assessment and grading of AG

All pathological diagnoses were made by histological examination of gastric biopsy samples (corpus, antrum and incisura) following the updated Sydney System [16]. We used 10% formalin to fix biopsies, and the samples were sectioned and stained by hematoxylin and eosin. Endoscopic atrophy was assessed by the Kimura-Takemoto classification [17]. The classification of gastritis was calculated by OLGA and OLGIM staging systems, in which a higher stage number represents a more severe lesion [15]. Two independent pathologists, who were blinded to patient characteristics, assessed the biopsies. The biopsies were assessed by a third pathologist again until agreement was reached to prevent disagreement.

### Determination of plasma folic acid, gastrin-17, homocysteine, pepsinogen I and pepsinogen II levels

Blood samples were obtained from 128 patients for the measurement. Gastrin-17, pepsinogen I, and pepsinogen II levels were determined with an ELISA kit. The absorbance of samples was measured at 450 nm. To get serum sample concentrations, assay results were analyzed by GastroSoft 1.51b for Excel (Biohit HealthCare). High-performance liquid chromatography was used to measure levels of homocysteine, and radioimmunoassay was used to measure plasma folic acid levels. In the study, hyperhomocysteinemia level was defined as a concentration more than 15.0 μmol/L and a concentration less than 6.0 ng/mL was regarded as folate deficiency.

### DNA extraction and genotyping of MTHFR polymorphism

We extracted genomic DNA from blood samples using a column extraction kit (QIAGEN Inc., USA). The DNA content was quantified using a Nanodrop spectrophotometer (BioLab). For MTHFR C677T genotyping, digital fluorescence molecular hybridization (DFMH) was performed using a commercial kit (Sino Era Genotech, Beijing, China) as described previously [18]. The gene polymorphisms were then analyzed with the real-time PCR (Tianlong, Xi’an, China) [19].

### Statistical analysis

Categorical variables were analyzed by percentages using χ^2^ test. Continuous variables were described by mean values with standard deviations and were compared between groups using Student’s t-test. Relationships between the clinical parameters were assessed by Spearman’s rank test. The agreement between endoscopic and histological findings regarding the classification of AG was analyzed based on the kappa value. If the *P*-value was less than 0.05, results were considered significant. Multiple comparisons were made using the binominal logistic regression analysis. The Statistical Package for the Social Sciences (SPSS Inc., USA) software version 25.0 was used for statistical analyses.

## Results

### *TT genotype is more frequent among younger AG patients without H. pylori* infection

**Fig. 1:**
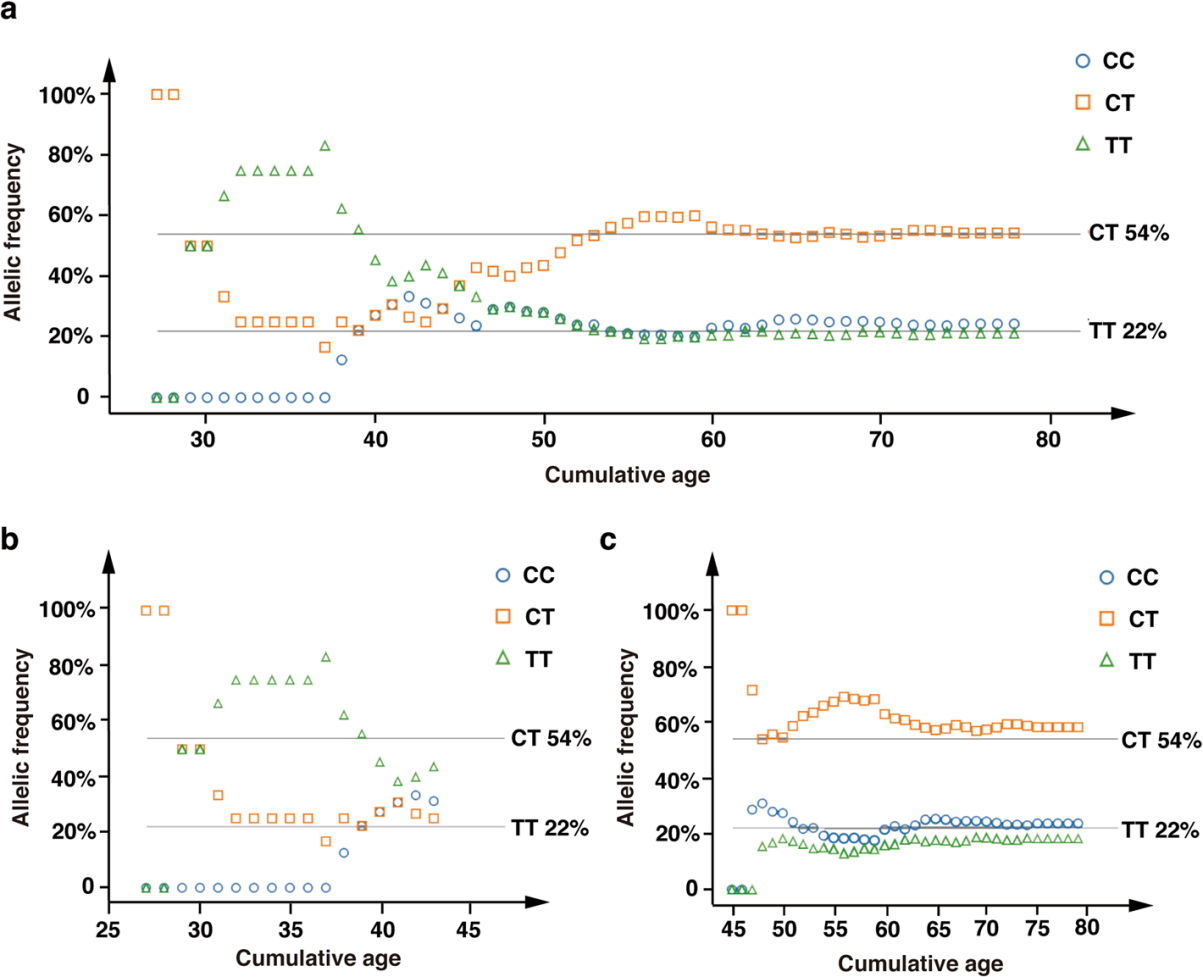
MTHFR C677T allelic frequency (Y-axis) with respect to cumulative age (X- axis) in different patient age groups. The MTHFR C677T allelic frequency in all *Helicobacter pylori*-negative patients included in the study. The frequency of the TT genotype was significantly higher among patients aged ≤44 years than among patients over 44 years (41.18% vs. 18.92%; *P* = 0.039). The two horizontal lines represent the genotype frequencies in 128 patients in our study (TT = 21.88%, CT = 53.91%). **b**. The MTHFR C677T allelic frequency in patients aged 27–44 years. **c**. MTHFR C677T allelic frequency in *Helicobacter pylori*-negative patients aged 45–80 years

The study group consisted of 128 AG patients (50.00% men, age range 27–80 years, mean age 55.1 ± 10.2 years). The clinical characteristics of patients are shown in Table 1. The genotypes and frequencies observed in our population were TT in 21.88% (28/128) of patients, CT in 53.91% (69/128), and CC in 24.22% (31/128). This distribution followed the Hardy-Weinberg equilibrium (*P* = 0.817). Generally, the allele frequencies of the MTHFR C677T genotypes should be stable for people of all age groups. However, in patients 44 years or younger (≤44 years), the frequency of the TT genotype was significantly higher than that in older patients greater than 44 years (41.18% vs. 18.92%; *P* = 0.039; Fig. 1). For the 17 patients aged 27–44 years, the MTHFR C677T genotypes and frequencies were TT in 41.18% (7/17), CT in 29.41% (5/17), and CC in 29.41% (5/17). In the 111 patients older than 44 years, the genotypes and frequencies were TT in 18.92% (21/111), CT in 57.66% (64/111), and CC in 23.42% (26/111). In addition, the pepsinogen I to pepsinogen II ratio (PGR) was significantly higher among patients aged 44 years and older compared to that among patients older than 44 years (13.0 ± 4.2 vs. 10.9 ± 3.9; *P* = 0.045).

**Table 1:** *Helicobacter pylori*-negative patients characteristics stratified by MTHFR C677T genotypes

Characteristic	CC *n* = 31	CT *n* = 69	TT *n* = 28	*P* Value
**Age (years), mean ± SD**	55 ± 10	56 ± 9	54 ± 13	N.S
27–44	5	5	7	
45–62	17	48	14	
63–80	9	16	7	
**Male, n (%)**	15 (48.4%)	34 (49.3%)	15 (53.6%)	N.S
**Family history of gastric cancer in first-degree relatives, n (%)**	7 (22.6%)	19 (27.5%)	4 (14.3%)	N.S
**Smoking status**				N.S
Never	23	50	19	
Current/Former	8	19	9	
**Alcohol status**				N.S
Never	23	53	19	
Current/Former	8	16	9	
**Gastrin-17 (pmol/L)**	8.2 ± 20.6	5.8 ± 11.4	3.8 ± 6.6	N.S
**Pepsinogen I (μg/L)**	104.8 ± 54.8	103.6 ± 69.5	81.1 ± 31.3	N.S
**Pepsinogen II (μg/L)**	10.5 ± 6.3	10.1 ± 8.6	8.4 ± 3.8	N.S
**PGR**	11.3 ± 4.7	11.4 ± 4.0	10.5 ± 3.4	N.S
**BMI (kg/m**^**2**^**)**	22.4 ± 2.8	22.3 ± 2.8	22.6 ± 2.5	N.S

*CC* MTHFR 677CC, *CT* MTHFR 677CT, *TT* MTHFR 677TT, *SD* Standard deviation, *PGR* Pepsinogen I and pepsinogen II ratio, *Hcy* Homocysteine, *BMI* Body mass index, *N.S* Not significant

The analyzed factors were age; gender; presence of peptic ulcers; smoking and drinking habits; body mass index; Hcy; BMI; family history; MTHFR C677T genotype. The number of each variable or mean ± SD, odd ratio, 95% confidence interval, and *P*-value are shown in Table 2. MTHFR C677T genotype and aging remained independent risk factors.

**Table 2:** Variables examined for determining the risk of moderate-to-severe lesions

Variables	Number	*P* Value	OR	95%CI	*P* Value
**Age (> 55/< 55 year)**	64/64	0.04	2.28	1.04–5.00	0.04
**Gender (male/female)**	64/64	0.85	1.07	0.40–2.82	0.90
**Peptic ulcer** **(positive/negative)**	21/107	0.66	1.33	0.56–2.76	0.59
**Smoking (yes/no)**	36/92	0.79	0.77	0.25–2.36	0.65
**Drinking (yes/no)**	33/95	0.75	1.32	0.43–4.00	0.63
**BMI (kg/m**^**2**^**)**	22.4 ± 2.7		1	0.86–1.16	0.99
**Hcy (μmol/L)**	12.8 ± 5.6		0.98	0.91–1.05	0.54
**Family history (yes/no)**	30/98	0.77	1.08	0.41–1.45	0.76
**MTHFR C677T (CC + CT/TT)**	100/28	0.01	4.12	1.29–13.21	0.02

*Hcy* Homocysteine, *BMI* Body mass index, *OR* Odds ratio, *P*-values were calculated using a binominal logistic regression analysis. *CI* Confidence interval

### *AG may be main cause of hyperhomocysteinemia in AG patients without H. pylori* infection *rather than MTHFR polymorphism*

As shown in Table 3, the mean levels of Hcy in patients with the CC genotype, CT, or TT genotypes were 11.7 ± 5.4 μmol/L, 12.9 ± 5.6 μmol/L or 13.5 ± 6.0 μmol/L, respectively. The highest levels of Hcy were observed in patients with TT genotype followed by those with CT and CC genotypes (*P* > 0.05). In addition, no significant difference was observed in the incidence of hyperhomocysteinemia (> 15 μmol/L) among patients with the different MTHFR C677T genotypes (*P* = 0.82). However, folic acid deficiency (≤6 ng/mL, as defined in ref. [20]) was observed more often in patients with TT genotype compared with the CT and CC genotypes (*P* = 0.001).

**Table 3:** Baseline folic acid and Hcy levels in AG patients without *Helicobacter pylori* infection stratified by MTHFR C677T genotypes

Characteristic	CC *n* = 31	CT *n* = 69	TT *n* = 28	*P* Value
**Folic acid (nmol/L)**	42.5 ± 12.6	36.9 ± 13.9	32.6 ± 17.7	N.S
**Hcy (μmol/L)**	11.7 ± 5.4	12.9 ± 5.6	13.5 ± 6.0	N.S
**Hyperhomocysteinemia**				
Yes	8 (25.8%)	22 (31.9%)	8 (28.6%)	N.S
No	23 (74.2%)	47 (68.1%)	20 (71.4%)	
**Folic acid deficiency**				
Yes	1 (3.2%)	9 (13.0%)	11 (39.3%)	0.001
No	30 (96.8%)	60 (87.0%)	17 (60.7%)	

*N.S* Not significant

As shown in Table 4, in our population, 29.69% (38/128) of AG patients had hyperhomocysteinemia and 16.41% (21/128) of AG patients had folic acid deficiency. We found that patients with folic acid deficiency had a significantly higher incidence of hyperhomocysteinemia compared with patients without folic acid deficiency (52.38% [11/21] vs. 25.23% [27/107], *P* = 0.013).

**Table 4:** Association between hyperhomocysteinemia and folic acid deficiency in AG patients without *Helicobacter pylori* infection

		Hyperhomocysteinemia		
		No	Yes	P
**Folic acid deficiency**	No	80	27	0.013
	Yes	10	11	

### Association between high-risk OLGA/OLGIM stages III-IV and MTHFR C677T polymorphism

The results regarding the influence of the MTHFR C677T polymorphism on lesion status in the gastric mucosa of AG patients are presented in Table 5. The antrum region showed the highest frequency of atrophy or IM (86.72%, 111/128), followed by the incisura (37.50%, 48/128) and corpus (8.59%, 11/128). We found no association between MTHFR C677T polymorphism and lesions in the corpus and antrum (*P* > 0.05). However, in the incisura part of the stomach, patients with TT genotype showed a higher susceptibility to develop lesions including atrophy or IM (CC + CT vs. TT: 40.00% [40/100] vs. 64.29% [18/28], *P* = 0.02). OLGA and OLGIM stages III-IV were observed more frequently in patients with TT genotype compared with the CC + CT genotypes (for OLGA: CC + CT vs. TT: 16.50% [17/103] vs. 44.00% [11/25], *P* = 0.003; for OLGIM: CC + CT vs. TT: 16.05% [13/81] vs. 31.91% [15/47], *P* = 0.036).

**Table 5:** Baseline features of lesion status in the gastric mucosa of patients with AG and without *Helicobacter pylori* infection stratified by MTHFR C677T genotype

	Genotypes				C allele dominance		
	CC *n* = 31	CT *n* = 69	TT *n* = 28	P	CC + CT *n* = 100	TT *n* = 28	P
**Lesions in biopsies**							
Incisura							
yes	10 (32%)	30 (43%)	18 (64%)	0.04	40 (40%)	18 (64%)	0.02
no	21 (68%)	39 (57%)	10 (36%)		60 (60%)	10 (36%)	
Antrum							
yes	28 (90%)	62 (90%)	21 (75%)	0.15	90 (90%)	21 (75%)	0.06
no	3 (10%)	7 (10%)	7 (25%)		10 (10%)	7 (25%)	
Corpus							
yes	1 (3%)	5 (7%)	5 (18%)	0.17	6 (6%)	5 (18%)	0.22
no	30 (97%)	64 (93%)	23 (82%)		94 (94%)	23 (82%)	
**Atrophy**							
Absent/Mild	19 (61%)	43 (62%)	12 (43%)	0.19	62 (62%)	12 (43%)	0.07
Moderate/Severe	12 (39%)	26 (38%)	16 (57%)		38 (38%)	16 (57%)	
**IM**							
Absent/Mild	14 (45%)	34 (49%)	6 (21%)	0.04	48 (48%)	6 (21%)	0.01
Moderate/Severe	17 (55%)	35 (51%)	22 (79%)		52 (52%)	22 (79%)	
**Moderate-to-severe lesions**							
Absent/Mild	11 (35%)	28 (41%)	4 (14%)	0.04	39 (39%)	4 (14%)	0.01
Moderate/Severe	20 (65%)	41 (59%)	24 (86%)		61 (61%)	24 (86%)	
**OLGA**							
I-II	26 (84%)	60 (87%)	17 (61%)	0.119	86 (86%)	17 (61%)	0.003
III-IV	5 (16%)	9 (13%)	11 (39%)		14 (17%)	11 (39%)	
**OLGIM**							
I-II	20 (65%)	48 (70%)	13 (46%)	0.162	68 (68%)	13 (46%)	0.036
III-IV	11 (35%)	21 (30%)	15 (54%)		32 (32%)	15 (54%)	

Lesions included atrophy or intestinal metaplasia; atrophy: atrophy located in any one biopsy; intestinal metaplasia: intestinal metaplasia located in any one biopsy; moderate-to-severe lesions: moderate to severe intestinal metaplasia, moderate to severe atrophy or low-grade intraepithelial neoplasia in any one location; CI: confidence interval

For moderate-to-severe lesions (moderate-to-severe IM, moderate-to-severe atrophy or low-grade intraepithelial neoplasia in any one location), TT homozygous patients were at an increased risk compared with CC + CT patients (*P* = 0.01). In addition, TT homozygous patients had an increased risk of IM at any location compared with CC + CT patients (*P* = 0.01). Although not statistically significant (*P* = 0.07), a trend towards a higher frequency of more severe atrophy at any location was observed in those with the TT genotype (CC + CT vs. TT: 38.00% vs. 57.14%).

The MTHFR C677T polymorphism was an independent predictor of the severity of lesions as shown in Table 5 (TT vs. CC + CT for atrophy: odds ratio [OR] = 2.18; 95% confidence interval [CI], 0.93–5.09; *P* = 0.07; for IM: OR = 3.39; 95% CI, 1.27–9.06; *P* = 0.02; for moderate-to-severe lesions: OR = 3.84; 95% CI, 1.24–11.90; *P* = 0.02; for OLGA: OR = 3.98; 95% CI, 1.54–10.23; *P* = 0 .004; and for OLGIM: OR = 2.45; 95% CI, 1.05–5.76; *P* = 0.039).

### Weak correlation between C-1/C-2 of endoscopic atrophy and OLGA stages I-II

**Fig. 2:**
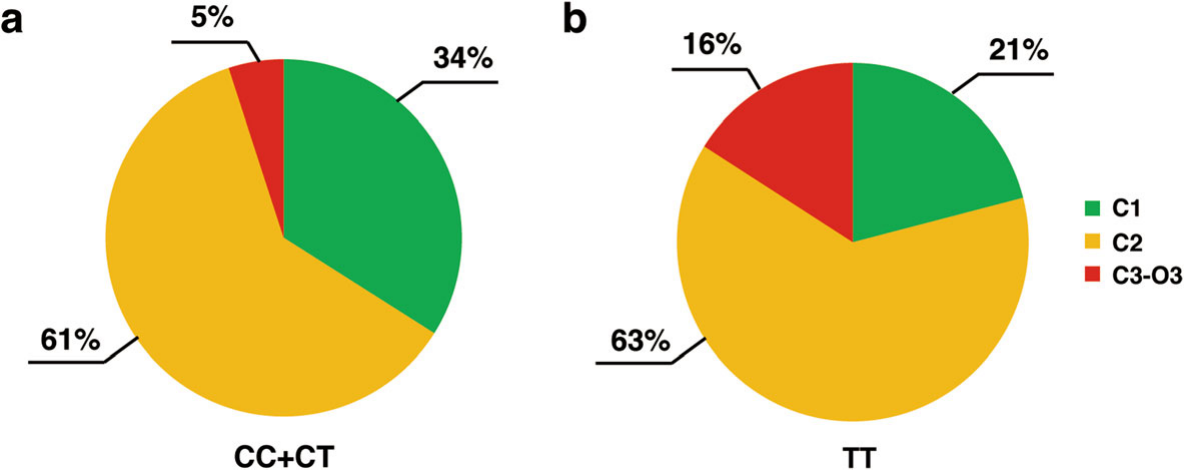
Distributions of patients with different Kimura-Takemoto endoscopic classifications among different MTHFR C677T genotypes. *Helicobacter pylori*-negative patients with TT genotype showed a trend toward a higher frequency of C-2 or C3-O3 lesions according to the Kimura-Takemoto endoscopic classification (CC + CT vs. TT: 66% vs. 79%, *P* = 0.29)

The Kimura-Takemoto classification (C-1, C-2, C-3, O-1, O-2 and O-3) has been performed in Eastern countries for the assessment and grading of AG [21]. In our study, the MTHFR C677T polymorphism was an independent predictor of the severity of lesions in patients stratified according to the OLGA and OLGIM systems. However, we found nothing statistically different on the severity of endoscopic gastric atrophy between those with the TT and CT + CC genotypes according to the Kimura-Takemoto endoscopic classification (*P* = 0.40, Fig. 2). In our study, according to the Kimura-Takemoto endoscopic classification, 92.59% patients were C-1 or C-2 and 80.47% patients of patients stratified according to the OLGA system were stages I-II. Based on these classifications, the strength of agreement between the C-1 or C-2 levels on endoscopic atrophy and OLGA stages I-II for the histological atrophy was fair, with a kappa value of 0.29 (95% CI, 0.06–0.50). In addition, correlations of C-1 or C-2 levels on endoscopic atrophy and stages I-II of OLGA were observed (Spearman’s rho = 0.31, *P* = 0.014).

## Discussion

DNA methylation is a pivotal epigenetic modification that can be altered in precancerous lesions [2]. As MTHFR is the key gene and metabolite in the one-carbon metabolism pathway that contributes to the provision of methyl groups and metabolism of homocysteine [22, 23], the MTHFR C677T polymorphism may be considered as a reliable factor for predicting the prognosis of gastric precancerous lesions [24, 25]. The reduced activity of the MTHFR enzyme resulting from TT mutation has been linked to aberrant DNA or RNA synthesis, repair, and chromosomal damage [6]. This study evaluated the degree of atrophy and IM in different biopsies to examine whether the TT genotype confers an increased risk for developing moderate-to-severe lesions (moderate-to-severe atrophy or IM in any one biopsy) in patients without *H. pylori* infection. In addition, patients with TT genotype were found to be at a higher risk of OLGA and OLGIM stages III-IV compared to patients with the CC + CT genotypes. It has been shown previously that OLGA stages I-II are associated with a lower risk while stages III-IV are associated with a higher risk of gastric cancer [26, 27]. Thus, in our study, the TT genotype was a risk factor for gastric precancerous lesions in patients without *H. pylori* infection. It is noteworthy to mention that conflicting results have been reported on the influence of the MTHFR C677T polymorphism on precancerous lesions or cancer. Some studies have shown an increased risk of gastric cancer development among Asians and Caucasians [12, 28], while others studies have reported a negative association [29, 30]. Conflicting results indicate that population-specific and geographical factors may account for this phenomenon. For example, the results in our study were based on the study group which included patients who were *H. pylori* negative. However, the study from Itou et al. was based on the study group which included both *H. pylori* negative and positive patients and did not examine the influence from the *H. pylori* infection [31]. In addition, the inconsistent results may be due to the methods of confirming the atrophic gastritis. In previous study, gastric atrophy was only evaluated with serum pepsinogens (PGI < 70 ng/dl and PGI/II < 3) while all pathological diagnoses were made by histological examination of gastric biopsy samples in our study [31]. It is well established that histological examination is still the gold standard. In studies using histological examination, MTHFR C677T genotypes can monitor stomach cancer risk among atrophic gastritis patients [32].

In addition, we should routinely include the incisura biopsies in sampling protocol for patients with TT genotype for further screening of gastric cancer risk. The incisura is the main lesion for the early-onset of atrophic-metaplastic evolution [33]. It may undergo more severe lesions than the antrum or corpus [34, 35].

A cross-sectional study showed an age-related trend with a growing prevalence of AG in people aged 35–44 years compared to those older than 44 years in Sweden [36]. The morbidity age for AG patients without *H. pylori* infection seems to be younger than previously thought. Previous studies suggested that the growing prevalence of overweight and obese patients resulted in this unexpected trend [36, 37]. In our AG population, we did not find such an association between the severity of AG and overweight or obesity (BMI shown in Table 1, *P* > 0.05). These observations in our study may be due to the fact that we did not establish a control group in the general population for comparison with AG patients, as was done in the study by Song et al. [36]. However, when we divided patients into two age groups (27–44 years and 45–80 years), the frequency of the TT genotype was much higher in the younger age group than in the older age group, indicating that AG patients with TT genotype might have a younger morbidity age and a longer duration of illness. As a result, AG patients with TT genotype may suffer from more severe gastric diseases. Previous studies have confirmed that aging is an independent risk factor for AG progression to gastric cancer [38]. In general population, the prevalence of AG in persons over 40 years is double that in those under 40 years [39]. In our study, however, the frequency of the TT genotype was lower in patients over 44 years of age. This may be due to some important transition of the dominant mechanism. Further research on the difference in MTHFR C677T genotype frequency in these two AG age groups is warranted.

Folate deficiencies may cause uracil misincorporation during DNA synthesis, which increases cancer risk [40]. The data from our study suggest that AG patients with TT genotype have a higher rate of folate deficiency compared with those with the CC + CT genotypes (*P* = 0.001), which will theoretically bring a higher rate of hyperhomocysteinemia. However, in our study, this was not the case. No significant difference was observed (*P* = 0.819), indicating that the AG may be more of a direct cause of hyperhomocysteinemia, which is in good agreement with previous research [11]. This phenomenon suggests that the AG factor may play a more important role in the presence of hyperhomocysteinemia than the MTHFR C677Tgenotype. As a result, AG patients are suggested to receive folic acid supplementation to reduce the risk of gastric cancer.

Although not statistically significant, patients with TT genotype in our study showed a trend towards a higher frequency of more severe lesions according to the Kimura-Takemoto endoscopic classification. In addition, some studies have reported that the severity of gastric atrophy assessed by the Kimura-Takemoto endoscopic classification is correlated with OLGA and OLGIM stages [21, 41]. In our study, however, the correlation was weak with a kappa value of 0.29.

To our knowledge, our study provides the first observation of an association between the MTHFR C677T polymorphism and gastric precancerous lesions in patients without *H. pylori* infection. We suggest that the TT genotype is associated with more severe lesions *in H. pylori* -negative patients. The biopsy of the incisura in AG patients with TT genotype will be useful for further screening of gastric cancer risk, especially for patients younger than 44 years. AG itself may be a contributing factor towards hyperhomocysteinemia. In addition, patients should be cautious about the potential risk of cardiovascular diseases in view of the association between hyperhomocysteinemia and vascular injury [42].

## Conclusions

Based on our findings, the effects of the MTHFR C677T polymorphism on gastric precancerous lesions have been systematically examined. We propose that MTHFR C677T genotyping could be useful in identifying *Helicobacter pylori*-negative patients at increased risk for moderate-to-severe atrophy or IM. Such screening may be valuable clinically in assessing the risk and prognosis of gastric precancerous lesions. In addition, AG patients should receive appropriate folic acid supplementation to prevent hyperhomocysteinemia. Further standardized research including well-designed and strictly implemented trials are required to confirm that the MTHFR C677T genetic polymorphism is an independent predictor of the severity of AG.

## Data Availability

The datasets used and/or analysed during the current study are available from the corresponding author on reasonable request.

## Abbreviations

MTHFR: Methylenetetrahydrofolate reductase
AG: Atrophic gastritis
*H. pylori*: *Helicobacter pylori*
IM: Intestinal metaplasia
OLGA: Operative Link on Gastritis Assessment
OLGIM: Operative Link on Gastric Intestinal Metaplasia Assessment
UBT: Urea breath test
Hcy: Homocysteine
